# Temperature-robust rapid eye movement and slow wave sleep in the lizard *Laudakia vulgaris*

**DOI:** 10.1038/s42003-022-04261-4

**Published:** 2022-11-29

**Authors:** Nitzan Albeck, Daniel I. Udi, Regev Eyal, Arik Shvartsman, Mark Shein-Idelson

**Affiliations:** 1 School of Neurobiology, Biochemistry, and Biophysics, Tel-Aviv University, Tel-Aviv, Israel; 2 Sagol School of Neuroscience, Tel-Aviv University, Tel-Aviv, Israel

**Keywords:** Experimental evolution, Circadian rhythms and sleep

## Abstract

During sleep our brain switches between two starkly different brain states - slow wave sleep (SWS) and rapid eye movement (REM) sleep. While this two-state sleep pattern is abundant across birds and mammals, its existence in other vertebrates is not universally accepted, its evolutionary emergence is unclear and it is undetermined whether it is a fundamental property of vertebrate brains or an adaptation specific to homeotherms. To address these questions, we conducted electrophysiological recordings in the Agamid lizard, *Laudakia vulgaris* during sleep. We found clear signatures of two-state sleep that resemble the mammalian and avian sleep patterns. These states switched periodically throughout the night with a cycle of ~90 seconds and were remarkably similar to the states previously reported in *Pogona vitticeps*. Interestingly, in contrast to the high temperature sensitivity of mammalian states, state switches were robust to large variations in temperature. We also found that breathing rate, micro-movements and eye movements were locked to the REM state as they are in mammals. Collectively, these findings suggest that two-state sleep is abundant across the agamid family, shares physiological similarity to mammalian sleep, and can be maintain in poikilothems, increasing the probability that it existed in the cold-blooded ancestor of amniotes.

## Abstract (web summary)

Analysis of sleep states in the rough-tail rock agama lizard suggests neurophysiological similarity between reptilian and mammalian two-state sleep features. In contrast to mammals, these features remain robust to the large temperature fluctuations experienced by reptiles.

## Introduction

Sleep is a fundamental behavior across the animal kingdom and is essential to normal animal function and survival^1,2^. This conserved behavior rely on the patterned activation of neuronal circuits throughout the brain. Yet, we still do not fully understand the functional role played by these activation patterns. A valuable strategy for generalizing principles of sleep function and their link to neurophysiology is by conducting comparative investigations across species^3,4^. This comparative approach has the added benefit of placing sleep within an evolutionary context, providing further explanatory power^5,6^. While behavioral characterization of sleep exists across vertebrate classes, the neurophysiological correlates of sleep have mostly been studied in mammals.

These electrophysiological investigations have revealed that sleep is characterized by two primary brain states: slow-wave sleep (SWS) characterized by high-amplitude low-frequency fluctuation in the EEG δ band (0.5–4 Hz), and rapid eye movement (REM) sleep, defined by low-voltage, high-frequency fluctuations^7^. Cortical δ waves are underlined by periodic switches between two excitability states: up states during which large populations are synchronously active, and down states with only sparse spiking^8^. Concomitant with thalamo-cortical SWS, the hippocampus engages in a similar shift between long epochs of sparse firing separated by sharp waves (ShW) during which activity is drastically elevated^9^, and exhibits oscillatory ripples (SWR) at 110–200 Hz^10^. Conversely, during REM sleep, overall firing rates increase, and activity is typified by more desynchronized firing patterns within both the cortex and the hippocampus^11,12^. These brain dynamics are accompanied by an additional set of physiological properties such as rapid eye movement, loss of muscle tone, muscle twitches, and elevated respiration rates^13,14^.

Sleep states are controlled by nuclei in the brain stem^7^, basal forebrain^15^, and the hypothalamus^16,17^. These areas have clear homologs across vertebrates and are tightly linked to thermo-regulation^18–21^. Recent studies report that cortical sleep patterns are also coordinated by higher brain areas within the pallium. Specifically, the claustrum has recently been shown to be an important coordinator of SW activity in mice, mediating cortical transitions to SWS^22,23^. While the details of this pallial coordinating network are not entirely understood, it is likely to play a major role in sleep functions such as memory consolidation ^24–26^.

**Fig. 1:**
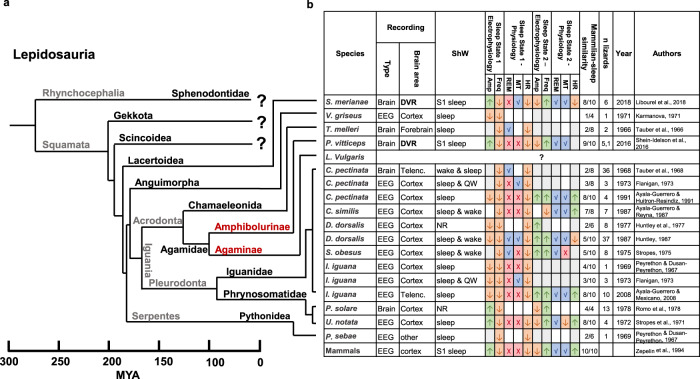
Comparative review of sleep studies in Lepidosauria. **a** Cladogram of Lepidosauria (based on ref. ^47^) including all squamate families in which sleep has been studied. Black font - Families, Red font – sub-families, gray font – order/suborder/clade. **b** Table summarizing the main electrophysiological and physiological features observed during behavioral sleep across studied species. The last row shows sleep features in mammals for comparison. Sleep state 1 features (increases and decreases) are relative to wakefulness, and sleep state 2 features are relative to sleep state 1 features. Mammalian similarity score was calculated as the fraction of similar features. Abbreviations: Amp signal amplitude, Freq signal frequency, REM rapid eye movements, MT muscle twitches, HR heart rate, ShW sharp waves, MYA million years ago. Color code: green up arrow – increase; orange down arrow – decrease; blue V – found; red X – not found; light gray – no change; white – not reported.

While the large majority of studies focused on mammals, the presence of sleep has also been well established in birds^27,28^ prompting researchers to hypothesize that such sleep patterns existed in stem amniotes, the common ancestors of mammals and birds. If this is the case, two-state sleep should also be found across reptiles. Indeed, several studies (mostly in the 1970s) conducted in non-avian reptiles, set to investigate this question^29^. Within this class, lepidosaurs are particularly interesting as they are the most distant from aves^30^. While these studies showed that behavioral sleep was abundant across lepidosaurs, evidence of electrophysiological sleep remained largely inconclusive (Fig. 1). All studies found a decrease in EEG frequency as animals entered a state of quiescence, however, EEG amplitude during this transition varied between studies^31–34^. Many reported only one EEG pattern during behavioral sleep, others reported two different EEG sleep patterns. Most of these reports named one of the patterns “quiet sleep”, defined by its similarity to quiet wakefulness, but lower in amplitude and frequency. The second reported pattern was usually very short, named “active sleep” and was defined by the similarity to EEG patterns during active wakefulness, e.g. high amplitudes and frequency. Note that these definitions are not identical to the ones used in current literature to describe behavioral sleep in young mammals^35,36^. Interestingly, the active sleep patterns were, in some cases, linked to one or more REM-like physiological patterns^37–42^. Two of the studies that showed two EEG patterns, reported that they were similar to mammalian SWS and REM in term of amplitude and frequency^43,44^. Taken together, insufficient and contradicting evidence in different species, and even in the same species^33,42,45^, led to ambiguity regarding the existence of two-state sleep in reptiles^29,46^.

Recent evidence from the lizard *Pogona vitticeps* tells a possibly different story on reptilian sleep. This study reported two periodically-alternating sleep states which bear high similarities to mammalian and avian states^48^. In contrast to previous studies, brain states in this study were found in the dorsal ventricular ridge (DVR). This may explain why these brain states were previously missed in most other studies that used EEG recordings from above the dorsal pallium. In addition, the authors used modern electrophysiological techniques and data analysis methods allowing them to track long-term LFP dynamics and identify slow oscillations, as well as to measure spiking patterns, thus revealing the underling organization of single cell population patterns. A follow up study revealed that the sources of SWR that occur during SWS in *P. vitticeps* originated from the reptilian homolog of the claustrum, suggesting sleep-control similarities between mammals and reptiles^49^.

These results were compared with recordings from another lizard species, the Argentine Tegu (*Salvator merianae*)^50^. While two states of electrophysiological sleep (defined by a transition in frequency profiles) were detected in the Argentinian Tegu, they lacked the high similarity with mammalian states and the alternating periodicity observed in *P. vitticeps*. The Tegu mostly exhibited a SWS-like brain state with transient activations of a REM-like state typified by 15 Hz oscillations appearing at the beginning and the end of the sleep period. Interestingly, both species exhibited ShW activity preferentially during the SWS-like state. Since the highly-structured sleep-state manifestation in *P. vitticeps* was never reported in any other species, it has hitherto remained unclear whether these mammalian-like sleep patterns are unique to *P. vitticeps* (possibly due to a specific adaptation) or whether they exist in other species. Furthermore, since only a limited number of physiological parameters associated with sleep transitions were reported in *P. vitticeps*, the functional homology between two-state sleep in *P. vitticeps* and mammals remains to be determined. Finally, if indeed brain state transitions exist in ectotherms, it is unclear how they can be robustly maintained given the high sensitivity of sleep states to temperature fluctuations reported in mammals^51^.

To answer these questions, we investigated sleep patterns in the rough-tail rock agama (*Laudakia vulgaris*, previously known as *Laudakia stellio*), a member of the Agamid lizard family. *L. vulgaris* and *P. vitticeps* belong to separate monophyletic groups that diverged more than 100 million years ago, making *L. vulgaris* a prime candidate for investigating two-state sleep across Agamids (Fig. 1^52^). *L. vulgaris* are diurnal, rock-dweller heliotherms. Behaviorally, these lizards sun-bask to control body temperature and tend to sit-and-wait for prey^53^. We found that *L. vulgaris* showed a prolonged period of behavioral sleep during the night with a clear division into two states with different spectral and amplitude profiles similar to mammalian SWS and REM sleep states. These states shifted periodically and showed a high abundance of SWR in the SWS-like state. Interestingly, we found that eye movements, breathing rate and the probability of muscle twitches increased predominantly during the REM-like state. Finally, sleep states transitions were observed across a wide range of temperatures and showed frequency scaling consistent with the Arrhenius equation confirming that they can be robustly maintained in poikilotherms.

## Results

### Behavioral sleep in *L. vulgaris*

**Fig. 2:**
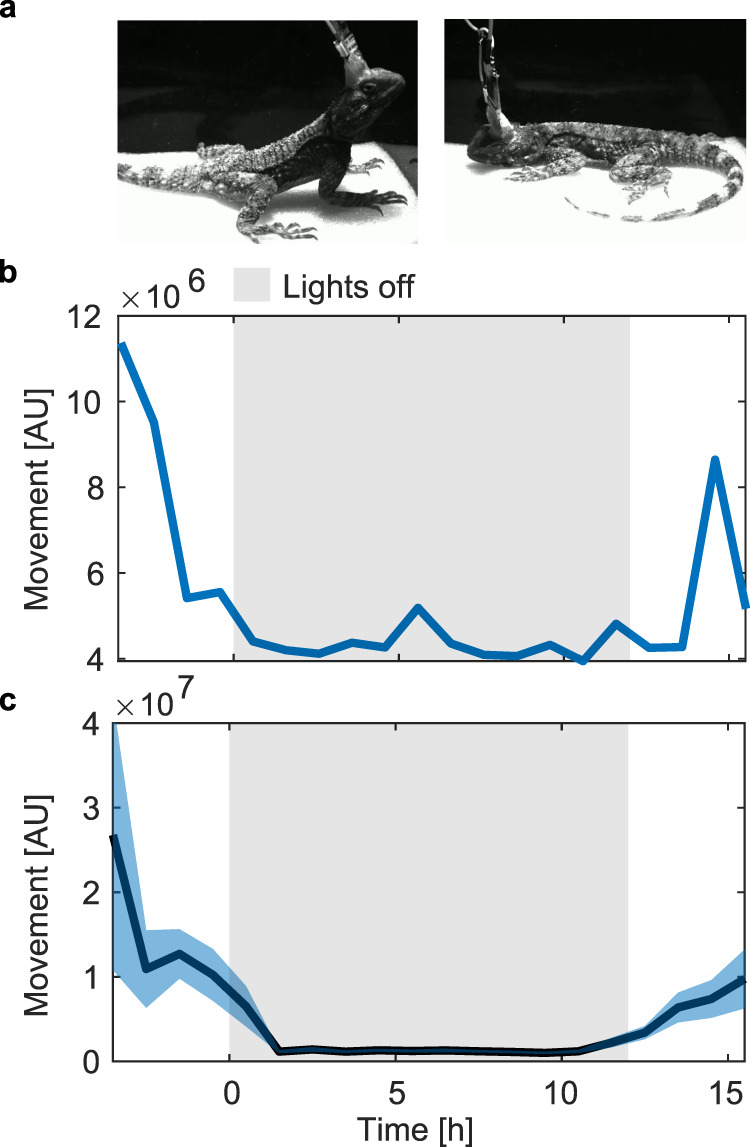
Long consecutive periods of behavioral sleep in *L. vulgaris*. **a** Images captured during wakefulness (left) and sleep (right). During sleep, the lizard assumes a typical sleeping posture with its head laying on the ground and its limbs spread to the sides of the body. Movement analysis surrounding the sleep period for one night (**b**) and averaged (1-h bins) over nights and animals (**c**). Average in black and standard error in blue shade. Gray shades mark the lights-off period.

We first examined the behavioral sleep patterns of wild-caught *L. vulgaris* under lab conditions. After a few days of accommodation to their home terrarium, the lizards showed clear entrainment to diurnal cycles (12/12 light/dark). Before the beginning of the lights-off period (19:00–7:00), the animals reduced their movement, lay down (assuming a typical sleep posture), and closed their eyes (Fig. 2a). To quantitatively evaluate movement around sleep time, we placed the lizards in the recording arena 3 h before the lights-off period and measured their movement using accelerometers (see Methods). The lizards gradually decreased their movement in anticipation of the lights-off period. Their movement levels remained low for the duration of the night and increased again in the morning (Fig. 2b). This movement profile was consistent across animals (Fig. 2c, 89 nights from 7 animals) and consistent with previous results from *P. vitticeps*^48^.

### Two-state neurophysiological sleep in *L. vulgaris*

**Fig. 3:**
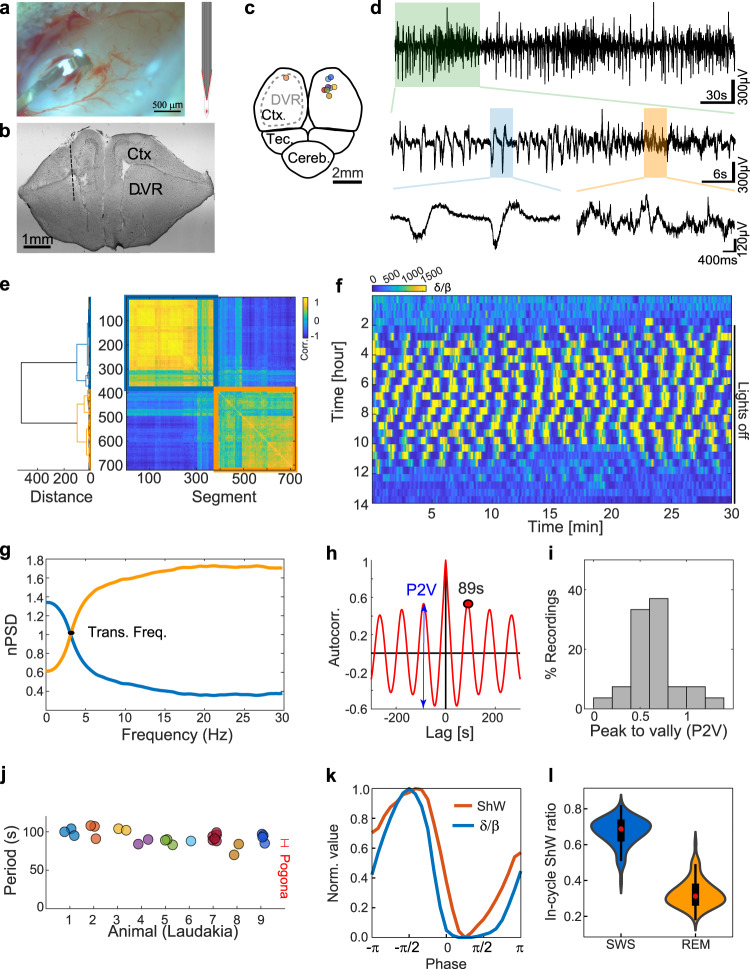
Sleep is composed of two periodically switching brain states. **a** Flex-probe implantation in DVR. A top image of the craniotomy shows the flexible probe inserted into the brain. The electrode layout of the 32-channel probe (200μm width) is shown on the right. **b** A nissl stained coronal section from implanted *L. vulgaris*. The dotted line indicates the presumed electrode track. **c** Probe implantation positions on a schematic top view of the brain. Insertion positions are marked by circles (each color corresponds to one animal). **d** Raw data traces recorded from the DVR during sleep. Top: A trace showing a few transitions between two distinct activity patterns. Middle: Zoom into a segment showing only one transition. Bottom: Zoom into two segments taken from each activity state. **e** Ordered correlation matrix between the spectral profiles of 700 consecutive (10 s long) recorded traces during sleep. A dendrogram based on Euclidian metrics and Ward linkage calculated on the correlation matrix is plotted on the left. A separation into two groups (orange and blue) is evident. **f** *δ*/*β* dynamics during sleep. The *δ*/*β* ratio measured over 10-s moving windows (1-s steps) around night-time. Each horizontal row represents a 30-min segment (running left to right); successive 30-min segments run continuously from top to bottom. This slow alternation (high versus low *δ*/*β*) starts shortly after the lights are turned off, and continues for the majority of the sleep (the lights-off period marked by a black line). **g** Normalized and averaged power spectral density of the data segments in each of the groups in (**e**). One group is typified by relatively higher frequencies and the other by relatively lower frequencies (colors correspond to clusters in (**e**)). **h** The autocorrelation function of the *δ*/*β* dynamics reveals a clear cycle with a period of 89 s (first peak, black dot). The difference between the first peak and the first valley (P2V) is marked by a blue arrow. 95% Confidence bounds (at *y* = ±0.024) are marked by black lines. Panels d-h were taken from the same night’s recording. **i** histogram of P2V values for all the recordings in (**j**). **j** The calculated cycle period, recorded at temperatures between 26–28 °C. Each circle corresponds to one night and each color to one animal. The period value range (±1std) recorded from *P. vitticeps* (taken from ref. ^48^) is shown for comparison. **k** The average phase of ShW rate (red) and *δ*/*β* (blue) during the oscillation cycle. **l** A violin plot of the normalized ShW rate distribution during REM-like and SWS-like phases, showing significantly increase in ShW rates during SWS-like state (red – median, box - 1st and 3rd quartiles, whiskers - non-outlier minimum and maximum).

After verifying behavioral sleep, we turned to characterizing the neurophysiological correlates of sleep. We implanted 32 channels, thin (4 µm thick) and flexible polyamide electrode arrays (Fig. 3a) in the anterior DVR (Fig. 3b, c) by lowering the highest electrode to a position 1 mm below the cortical surface. This choice was motivated by the robust manifestation of sleep states^48^ and the proximity to the source of ShW^49^ in *P. vitticeps*. Raw data traces recorded from the DVR revealed two distinct activity patterns (Fig. 3d). One pattern (Fig. 3d, blue shade) was characterized by high-amplitude ShW, and the second by lower amplitude fluctuations (Fig. 3d, orange shade). To quantify this division into states, we analyzed the spectral profiles during a 2-h interval of sleep. We first divided the recording into consecutive 10 s segments and calculated the spectral context of each segment. We next correlated the spectral context between all segment pairs and clustered them using a dendrogram algorithm. This analysis revealed a clear separation to two different spectral profiles (Fig. 3e): one state with relatively high power in lower frequencies (Fig. 3g, blue trace) within the δ band (0–4 Hz), and one with predominantly greater power in higher frequencies (Fig. 3g, orange trace) within the β band (10-40 Hz). The transition frequency between these states was at ~4 Hz (Fig. 3g).

To track the dynamics of sleep states across the night, we calculated the ratio between the δ and β bands for the entire recording (Fig. 3f). We observed oscillations in the *δ*/*β* ratio beginning shortly after the dark period and ending after several hours. To extract the periodicity of these oscillations, we calculated the autocorrelation function of the *δ*/*β* time series. The oscillation period was 89 s (Fig. 3h). This value slightly varied across nights and between animals (Fig. 3j, 92.8 ± 9.5, mean ± std, 27 nights in 9 animals). To quantify the robustness of the oscillation, we calculated the peak to valley (P2V) difference in the cross-correlation function. The calculated P2V values for all recordings were high (Fig. 3i, avg. P2V = 0.65 ± 0.25) and well above the confidence bound for significant autocorrelation value (defined as zero), suggesting clear oscillations for all recorded nights. To investigate the ShW dynamics during these two states, we next identified ShW using a matched filter (Fig. S1a, b) and monitored their dynamics throughout our recordings. ShW rate gradually increased following the onset of sleep and decreased again towards the onset of wakefulness (Fig. S1c). ShW rate was high during the entire night and showed a clear locking to the *δ*/*β* cycle (Fig. 3k). This difference in ShW rate between states was highly significant (*p* value < 0.001) when pooling across animals and nights (Fig. 3l, 14,999 sleep cycles from 50 nights in 9 animals, *t*-test, *p* value = 1.5e^−22^). ShW were accompanied by a transient increase in high-frequency oscillations (60–200 Hz) occurring preferentially during the trough of the ShW (Fig. S1d–g). While such an increase was not observed in all recorded animals, it was most prominent in good recordings with high total power (Fig. S1h). Taken together, these findings constitute clear evidence of two-state sleep in *L. vulgaris* with characteristics that are remarkably similar to the REM and SWS states found in the lizard *P. vitticeps*, that likely diverged from the *L. vulgaris* more than 100MYA ^47,52^.

**Fig. 4:**
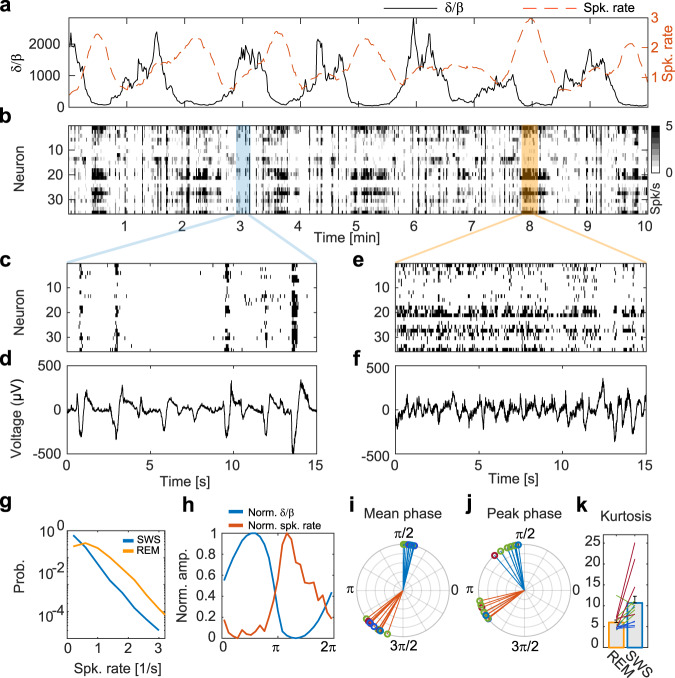
State transitions are underlined by changes in neuronal firing rates and synchronization patterns. **a** *δ*/*β* dynamics (black) overlaid on spiking rate dynamics (red) in the DVR. The spiking rates and *δ*/*β* amplitudes show inverse trends. **b** A raster plot of neuronal activity during the same recording segment as in (**a**). The spiking rate is color coded from white to black. Zoomed in time epochs of raster plots (**c**, **e**) and corresponding LFP traces (**d**, **f**) recorded during SWS (**c**, **d** - blue) and REM (**e**, **f** - orange), respectively. Notice the strong transient synchronized nature of activity during SWS (**c**, **d**) and the relatively tonic activity during REM (**e**, **f**). **g** The distribution of the spiking rate over one night. Spiking rates distributions during SWS are skewed towards lower values with a higher number of low rate events characteristic of silent periods. **h** The normalized (between 0 and 1) averaged spike rate and *δ*/*β* dynamics over all sleep cycles (*N* = 329) during one night. Notice that firing rate increases sharply as *δ*/*β* decreases. Average mean phase (**i**) and phase at peak (**j**) for *δ*/*β* (blue) and spike rate (red) for different nights and animals. A random phase component (std = 0.1rads - smaller than the bin size) was added to the location of the peak phase to visualize different nights. **k** Kurtosis values for spiking firing rate distributions during REM vs SWS for different nights and animals. SWS is typified by higher kurtosis values corresponding to bursting activity patterns. Error bars are standard errors. Circles (in **i**, **j**) and lines (in **k**) correspond to different animals color coded as in Fig. 3j.

We next sought to examine the spiking patterns underlying the recorded LFP signals. Recording single units for long periods of time is a challenge in reptiles, because of the relatively large gap between the brain and the cranium. Still, we were able to measure spiking activity in four animals for long periods post-implantation. Spiking activity was tightly locked to the *δ*/*β* dynamics (Fig. 4a, b). The spiking rate markedly increased during REM-like and decreased during SWS-like states. To quantify these dynamics, we first segmented the sleep period to REM-like and SWS-like epochs using phase analysis based on the Hilbert transform (Fig. S2) and then calculated firing rates for each state. Overall, the distribution of spike rates during the SWS-like state was skewed towards lower values relative to the REM-like state (Fig. 4g). This increase was steepest at the onset of the REM-like state, as evident from averaging rates across all sleep cycles (Fig. 4h). Thus, while the spiking rate is maximal during the REM-like state (low *δ*/*β*), its increase starts already during the SWS-like state together with the decline in *δ*/*β*, and it reaches a maximum shortly after the REM-like state onset (Fig. 4j and S2). Correspondingly, *δ*/*β* reached its average and peak phases at 0.45 ± 0.02π and 0.59 ± 0.06π radians, respectively, while spiking activity reached its average and peak phases at 1.29 ± 0.05π and 1.15 ± 0.03π radians, respectively (Fig. 4i, j, 14 nights in 4 animals).

The spiking population dynamics between states corresponded to differences between LFP profiles. During the SWS-like state, spiking activity was sparse but synchronized within short and intense neural activation time windows. These activation epochs were separated by longer periods of relative quiescence (Fig. 4c) and were well aligned to the times of SWR occurrences (Fig. 4d). In contrast, during the REM-like state, spiking activity was more tonic with spontaneous spikes constantly occurring (Fig. 4e). Correspondingly, firing rates were more evenly distributed over a wider range as indicated by the kurtosis of firing rates distributions (14 nights, 4 animals, Fig. 4k). These spiking patterns during the REM-like state were in line with LFP profiles characterized by higher frequencies (Fig. 4f, 3g). While activity was more tonic relative to SWS-like state, network spiking rates were not uniform over time and exhibited constant fluctuations aligned with fluctuations in LFP signals (Fig. 4f).

### Eye movement, breathing and muscle twitches are synchronized with sleep states

**Fig. 5:**
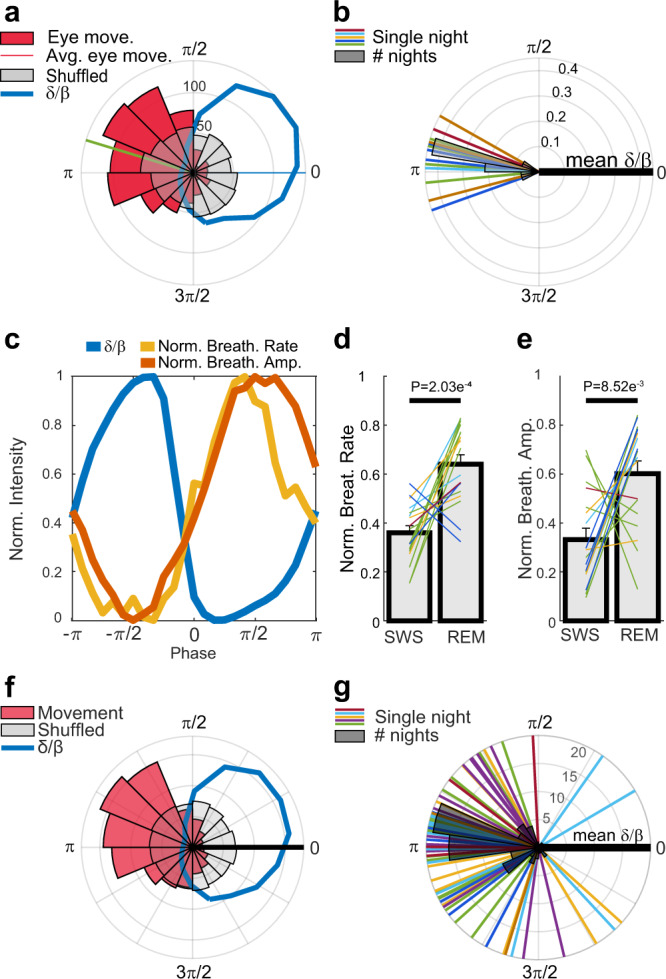
Physiological attributes are locked to sleep states. **a** Polar histogram of eye movement events (red) across sleep cycles compared to the normalized *δ*/*β* amplitude (blue) for all sleep cycles recorded for one night. All phases are relative to the *δ*/*β* mean phase (blue line at 0). Eye movements preferentially occur during REM-like sleep (low *δ*/*β*). A histogram of randomized movement times is shown in gray. **b** The average movement phase (green line in (**a**)) relative to the mean *δ*/*β* phase. Each line corresponds to the phase difference for one night and dark-gray polar bars are the histogram over all nights. **c** The normalized (0–1) averaged amplitude of the breathing rate (yellow), breathing amplitude (red) and *δ*/β (blue) across all oscillation cycles during one night. **d** Mean normalized breathing rate during REM-like vs SWS-like states. **e** Mean normalized breathing amplitude during REM-like vs SWS-like states. **f** Polar histogram of movement phases (red) and normalized *δ*/*β* amplitude (blue) for all sleep cycles recorded during one night. All phases are relative to the *δ*/*β* mean phase (black line at 0). Notice movements preferentially occur during REM-like sleep (low *δ*/*β*). A histogram of randomized twitch times is shown in gray. **g** The average movement phase relative to the mean *δ*/*β* phase. Each line corresponds to the phase difference during one night and dark-gray polar bars are the histogram over all nights. Error bars in (**d**) and (**e**) are standard errors. Color code in (**b**, **d**, **e**, **g**) as in Fig. 3j.

Previous studies have shown that eye movements in *P. vitticeps* are locked to REM^48,50^. We examined if such locking occurs also in *L. vulgaris* and whether there are additional physiological features that are associated with specific brain states during sleep. For this analysis, we only included recordings with robustly detectable state transitions (P2V > 0.2). Having defined REM-like/SWS-like state onsets and offsets (Fig. S2), we next identified (using an optic flow algorithm, see Methods) eye movements from video recordings during sleep. This analysis revealed that eye movements are tightly locked to the *δ*/*β* oscillations and occur mostly during the REM-like state (Fig. 5a, Video S1). This locking was consistent across nights and animals (14 nights, 5 animals, Fig. 5b). Since respiration has been linked to cortical activity in both mammals^59^ and lizards^60^, we used video recordings to track the movements of the ribcage area, which is a proxy for respiration dynamics during sleep (Fig. S3, Video S2). The region of the ribcage was manually marked at recording onset and tracked using the Kanade-Lucas-Tomasi algorithm to extract breathing rate. This analysis indicated that during the REM-like state (low *δ*/*β*), the breathing rate and amplitude increased compared to the SWS-like state (Fig. 5c). These increases were consistent across nights and animals (18 nights, 5 animals, Fig. 5d, e, *t*-test, *P* values: 2.03e^−4^ and 8.5e^−3^ correspondingly). Next, we analyzed recordings in which we acquired accelerometer data and could identify micro-movements (Fig. S4). Our analysis revealed that micro-movements were locked to the *δ*/*β* oscillation cycles and increased predominantly during the REM-like state (Fig. 5f). This increase was consistent across animals and nights (75 nights, 7 animals, Fig. 5g).

Head movements may be affected by or conjugated with multiple movement sources. To assess the interaction between head micro-movements, eye movements and breathing movement, we performed correlation and co-occurrence analyses (Supplementary Methods and Fig. S5). We found that head micro-movements and eye movements were significantly correlated (Fig. S5a, b). However, the majority of detected eye movements did not co-occur with head movements and vice versa (Fig. S5c, d). Since this co-occurrence may have resulted from eye movements detected by the head accelerometers, we examined the correlation between eye movement and head movement amplitudes. This correlation was not significant (*P* value > 0.05) for 83% of the recordings. Breathing was also significantly cross-correlated with head movements, yet, the maximal cross-correlation value in all recordings was very low (<0.02), indicating that these two movement types are largely independent during sleep (Fig. S5e–g).

### Sleep states across temperatures

**Fig. 6:**
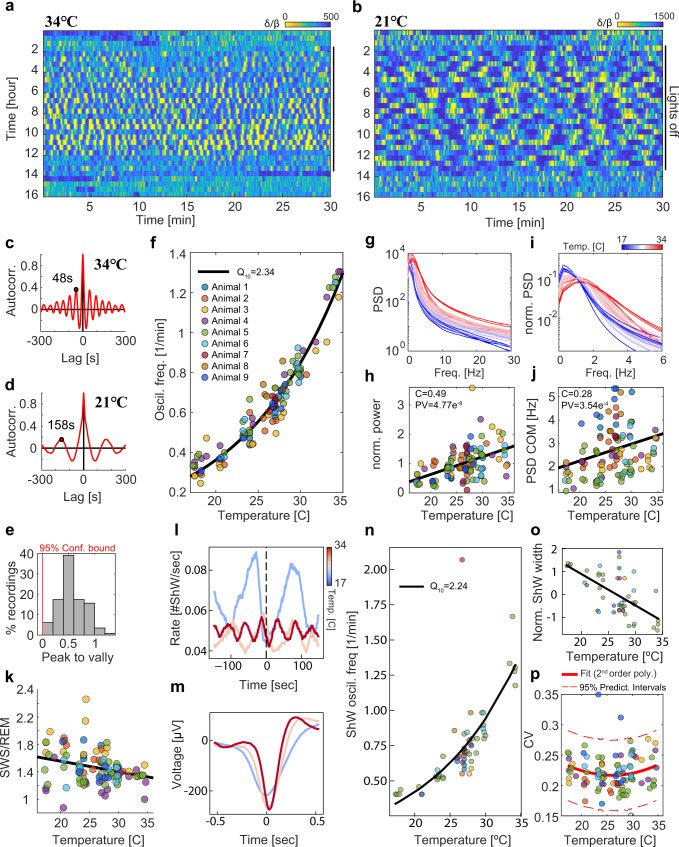
Two-state sleep is robust to temperature changes. **a**, **b** *δ*/*β* dynamics during single sleep nights (as in Fig. 3f) with (**a**) high and (**b**) low ambient temperatures (34 °C and 21 °C, respectively). **c**, **d** Autocorrelation functions for the nights in (**a**, **b**), respectively. Notice that a 13 °C temperature change corresponds to a more than 3-fold increase in the oscillation period (48 s vs 158 s). **e** Distribution of P2V values (see Fig. 3i) for all recorded nights. Peak autocorrelation values were all higher than the 95% confidence bound (at 0) indicating robust oscillations for all measured temperatures. **f** The *δ*/*β* oscillation frequency pooled over all nights. Fitting the data to the function *F* = *F*_0_*Q*_10_ (*T* − T_0_)/10 (black line) resulted in a *Q*_10_ coefficient of 2.3. **g** Averaged power spectral density (PSD) extracted from nights measured in different temperatures (color coded) from one animal. **h** PSD sum divided by the average sum over all nights of each animal and plotted for all animals. Notice positive correlation as a function of temperature (*C* = 0.49, *t*-test, *p* value = 5e^−8^). **i** Zoom into the plot in (**g**). Notice the shifts to higher frequencies. **j** Center of mass (COM) of the normalized PSD (**i**) for all nights. Notice positive correlation with temperature (*C* = 0.28, *t*-test, *p* value = 4e^−3^). **k** The ratio between SWS duration and REM duration for all nights. Notice negative correlation with temperature (*C* = −0.28, *t*-test, *P* value = 4e^−3^). **l** Average ShW rate around the SWS → REM transition (dashed vertical line, *t* = 0) for 3 different nights with temperatures 18 °C, 27 °C, 35 °C (color coded) recorded from one animal. **m** Average ShW waveform (for the nights in **l**). Notice changes in width. **n** The ShW oscillation frequency pooled over all nights. Fitting as in (**f**) (black line), *Q*_10_ = 2.26. **o** Average ShW width (full width at half maximum), Z-scored for all recordings of each lizard separately, pooled over all nights. Notice the negative correlation with temperature (C = −0.62, *t*-test, *P* value = 2e^−6^). **p** Coefficient of variation of sleep cycle duration as a function of temperature. Lines fitted to second-order polynomials. In panels (**f**, **h**, **j**, **k**, **n**, **o**, **p**), different circles correspond to different nights, and different colors to different animals (color coded as in Fig. 3j).

In mammals, sleep states are temperature-sensitive and switch following a temperature perturbation of a few degrees^51,61^. Therefore, we examined how temperature affects neural states in a poikilotherm experiencing a wide range of temperatures. To examine this, we increased the ambient temperature to 34 °C and measured brain dynamics during sleep. At this temperature, clear oscillatory activity was evident (Fig. 6a), but the period of oscillations shortened to 48 s (Fig. 6c). In contrast, when we reduced the temperature to 21 °C, the oscillation period dramatically increased to 158 s (Fig. 6b, d). To systematically study the oscillation temperature scaling properties, we exposed the lizards to recording nights with ambient temperatures ranging from 17°–35 °C. Interestingly, the lizards were able to preserve oscillatory dynamics across the entire temperature range and maintain clear transitions between the REM-like and the SWS-like states as indicated by the distribution of P2V (Fig. 6e). Plotting the frequency of oscillations as a function of the temperature for different recordings revealed a clear trend according to which oscillation frequencies increase with temperature increases (Fig. 6f). Assuming an exponential dependence of the oscillation rate on temperature (Arrhenius equation), we fit the data with ${R}_{T}={R}_{T0}{Q}_{10}^{(T-T0)/10}$, where *R*_T_ is the oscillation rate (cycles/min) at temperature *T*, *T*0 = 17 °C, and *Q*_10_ is the temperature coefficient^62^. This fit revealed a *Q*_10_ coefficient of 2.3 (Fig. 6f). We also observed that the transition frequency (as calculated in Fig. 3g) between the SWS-like and the REM-like state increased with temperature and fitted a *Q*_10_ coefficient of 2.0 (Fig. S6).

Previous studies have shown that neural tissues are more excitable in higher temperatures^63^. We therefore monitored LFPs (a proxy for synaptic currents and excitability) for different frequencies (0–30 Hz range corresponding to the *δ* and *β* frequency bands) across different nights. We observed that the total power in the frequency domain (corresponding to amplitude fluctuations in the time domain) increased with temperature (Fig. 6g). To quantify this, while considering the variability in recording quality (and LFP amplitudes) between animals, we first normalized the power by the mean power (across all temperatures recorded from a given lizard) and then calculated the correlation between the normalized power and temperature for all animals (Fig. 6h). This correlation was positive and consistent across the population (correlation coefficient = 0.49, *t*-test, *P* value 5e^−8^), despite expected changes in the tissue-electrode coupling over weeks of recording. In addition to the general increase in power, a clear shift towards higher frequencies, including the peak frequency, was observed in higher temperatures (Fig. 6i). Calculating the center of mass across the power spectra also revealed a significant increase with temperature (Fig. 6j, correlation coefficient = 0.28, *t*-test, *P* value 4e^−3^). Thus, sleep oscillations were preserved despite changes in tissue excitability. Finally, we examined if the relative duration of SWS-like and REM-like sleep changed for different temperatures (Fig. 6k). In general, the SWS-like state occupied a higher fraction of the sleep period and this fraction slightly decreased as the temperature increased (C = −0.28, *t*-test, *P* value = 4e^-3^).

We next examined how ShW dynamics are affected by temperature. The ShW rate fluctuated periodically and was locked to the *δ*/*β* cycles (Fig. 6l). The ShW oscillation period increased with temperature (Fig. 6l, n) and was fitted with a Q_10_ coefficient of 2.24. The ShW duration was also affected by temperature. ShW width markedly decreased with temperature (Fig. 6m) and Z-scoring the widths per animal showed a significant correlation with temperature across nights (Fig. 6o, Correlation coefficient = −0.62, *t*-test, *P* value = 2e^−6^). Furthermore, ShW amplitude increased with temperature, unlike the ShW widths (Fig. S7). This analysis suggests that as temperature increases, population activity during ShW becomes more synchronized (higher firing rates during shorter time windows). Although we observed significant oscillations across all measured temperatures, the robustness of the oscillation period across cycles (quantified by the robust coefficient of variation, CV) was mildly dependent on temperature. Overall, CV was better fitted with a second-order parabolic equation relative to a linear fit (*P* value of 0.0197 vs 0.8450, respectively based on an *F*-test on the model, fitnlm Matlab), indicating the possible existence of an optimal sleep temperature (Fig. 6p).

## Discussion

In this work, we demonstrated the existence of two sleep states resembling REM and SWS in the lizard *L. vulgaris*. These states are sustained for several hours during the night and switch periodically with a cycle of ~90 s (Figs. 2, 3). State switches are marked by changes in LFP frequencies and corresponding changes in the organization of population spiking patterns in the DVR. The SWS-like state is characterized by sporadic firing periods interrupted by short epochs of elevated synchronized activity, whereas the REM-like state is typified by overall higher and more sustained firing rates (Figs. 3, 4). We also found that eye movements, muscular twitches and increased respiration were preferentially locked to the REM-like state, whereas ShWs transiently increased during the SWS-like state (Figs. 3, 5). These brain activity profiles and associated physiological parameters are highly reminiscent of the sleep states found in mammals and birds^64–67^. We henceforth refer to these states as REM sleep and SWS. Finally, state switches were robust to ambient temperature fluctuations: increases in temperature resulted in increases in switching frequency while conserving the periodic transitions between states (Fig. 6).

The neurophysiological characteristics of sleep states we measured in wild-caught *L. vulgaris* were remarkably similar to the ones reported in *P. vitticeps*. These two species belong to separate sub-families of Agamid lizards that diverged more than 100 million years ago (Fig. 1^47,52^). Yet, both are characterized by very similar spectral sleep profiles with the transition between profiles occurring at ~4 Hz (Fig. 3g, S6^48^). Furthermore, in both species, states oscillated periodically for several hours during behavioral sleep with a very similar oscillation cycle (85 s in *P. vitticeps* vs 93 s in *L. vulgaris*). Finally, in both species, SWS was composed of long periods of sporadic low firing interrupted by transient increases in DVR spiking activity that appear as SWR in the LFP traces. REM sleep in both species was characterized by increased and tonic spike rates corresponding to the high-frequency-low-amplitude LFP traces (Fig. 4).

The high degree feature conservation between Agamid lizards is even more striking in light of the high inconsistency in neurophysiological sleep properties reported in past studies across reptiles (Fig. 1^29^). For example, the lizard *Ctenosaura pectinata* was studied by three different research groups, each reaching a different result^33,42,45^. Some of these inconsistencies may be the result of differences in experimental strategy and recording location. The majority of previous studies were conducted using EEG recordings from above the pallium in variable locations but not directly from the DVR (Fig. 1). The choice to record from the pallium was probably motivated by the homology between reptilian and mammalian dorsal cortices^68,69^. In contrast, in our study and those from *P. vitticeps*, sleep states were recorded from the anterior part of the DVR, and could not be detected robustly from dorsal cortex (and therefore also not from EEG recording from above the pallium).

This straightforward explanation is complicated by a recent study that used the same recording methodology in both Argentinian Tegu and *P. vitticeps*. The authors of this study replicated the sleep profile in *P. vitticeps* but did not find similar states in the Argentinian Tegu. However, only LFP data was acquired in this study, preventing the identification of changes in population spiking patterns. Indeed, while we found that LFP signals were good indicators of the underlying spiking patterns (Fig. 4), this may not always be the case as LFP patterns change depending on neuroarchitecture and the organization of return currents^70^. In addition, it is possible that Agamid lizards have particularly strong transients in population patterns, and that such transients are subtler in other lizards, making them harder to detect with LFP recordings. Good electrode-tissue coupling is critical for detecting small transitions in synchrony and such coupling is drastically reduced in lizards with large gaps between the brain and the skull due to increased brain movement^71,72^, as is the case in the Argentinian Tegu^50^. Correspondingly, our LFP sleep signatures were most prominent in recordings with clear spiking activity indicating good tissue-electrode coupling. Thus, further research is needed to determine whether technical issues can explain between-species differences, or whether there is large diversity in the sleep manifestation in lizards.

While our results strengthen the hypothesis that REM sleep and SWS existed in the common ancestor of mammals and reptiles, the homology of such states could also be the result of convergent evolution in mammals, aves and a few non-avian reptiles^29,48,73^. Our findings clarify that oscillatory two-state sleep shifts are widespread and strongly conserved across, at least, agamid lizards. However, further characterization of LFP and spiking patterns in DVR across other lizard families (Fig. 1) is required to determine if REM and SWS are more widespread than previously thought and therefore more likely to be homologous. Alternatively, we would have to explore the cognitive similarities between mammals, aves and Agamids that could explain such a strong convergent evolution. Such exploration could lead to a better understanding of sleep function across species.

We found that both eye movements and head movements were locked to REM. Most of these movements were independent, but some were temporally synchronous. One possibility is that these synchronous events resulted from eye movement being detected by the head accelerometers. This is unlikely however, given that during synchronous movements, the intensity of eye movement was not significantly correlated with the intensity of head movements. Thus, it is more likely that synchronous movements are part of physiological events that are synchronously activated during REM.

We found that, similarly to mammals, changes in respiration are coupled to sleep states. In humans, respiration rates increase and become irregular during REM sleep^59,74,75^, yet this increased breathing rate is correlated with lower breathing amplitude, resulting in lower respiration volume^76^. Interestingly, this reduction in respiration volume is accompanied by fast metabolic activations: an increase in cerebral O_2_^59,77^ and glucose metabolic rates^78^, increased ATP usage^79^, increased blood flow^78^ and increased glutamatergic activation^78,80^. In *L. vulgaris*, on the other hand, there is an increase in both breathing rates and breathing amplitude during REM (Fig. 5d,e). This increase in breathing volume fits the increased firing rates we measured during REM (Fig. 4), but is contrary to the reduction in mammalian respiration volume. This difference between mammals and reptiles could be related to the REM-induced muscle atonia in mammals^81^, which was only reported in two Squamata studies^29^, and absent from most other reports^41–45,50^. Consistent with this, a recent study in rats showed that the recruitment of the expiratory abdominal muscles during REM increases the respiration volume and reduces breathing variability (despite basal muscle atonia)^82^. This can explain how the lack of muscle atonia in reptiles can result in higher respiration volumes. The functional importance of this difference in breathing during sleep remains unclear. Nonetheless, it seems that eye movements, breathing changes and twitches are a part of a comprehensive physiological trend observed during REM, further strengthening the similarity between patterns in mammals and Agamid lizards.

The similarities between brain states in mammals and Agamids are surprising given that reptiles experience large circadian and seasonal fluctuations in their brain temperature. In mammals, slight changes in the brain temperature result in dramatic changes in the organization of neuronal dynamics during sleep^83^. Cooling the cortex of sleeping mice by 5 °C impaired the down states of slow oscillations and shifted neurons to persistent activity patterns reminiscent of REM or wakefulness^61^. Heating the thalamus by only 2 °C, significantly increased the frequency of sleep spindles^51^ whereas increasing the hippocampal temperature by 3 °C significantly changed the peak frequency, rate, and width of ShW^84^. These findings highlight the temperature sensitivity of the mammalian brain.

In birds, which are also endothermic, temperature manipulations seem to keep the coding scheme intact. Long et al. found that cooling the HVC nucleus by 5–10 °C slows the mating song speed of male zebra finches while preserving its general structure^85^. Correspondingly, natural changes in brain temperature explain faster female-directed singing (relative to non-directed singing) and song-tempo fluctuations during the day^86^. Such a temperature scaling scheme, conserving specific neural activity properties, aligns with our results in *L. vulgaris* (Fig. 6). Like the song structure in zebra finch, the switch between two activity patterns is conserved even as the transition rate changes. However, the power of this comparison is limited as it involved different brain circuits with different functions in birds and reptiles. Resolving these issues awaits measurements of brain state changes as a function of temperature in birds. Such data will also help determine whether the spiking patterns in birds are conserved despite population organization changes or whether information in specific areas (e.g. HVC) is state-independent. Alternatively, it may be the case that birds inherited a set of temperature regulating mechanisms that were lost in mammals. To resolve this question, data from an amphibian out-group will be needed.

The temperature robustness of *L. vulgaris* sleep highlights the importance of the two-state activity organization. But how can this organization be maintained and regulated? This question was investigated in the stomato-gastric ganglion of the Jonah crab (*Cancer borealis*). This crab exhibits a pyloric rhythm whose frequency increases 4-fold with temperature (7–31°C)^87^. Despite this increase, the phase relations between circuit components remained constant^88^. Interestingly, modeling work indicates that keeping this temperature robustness is challenging since neural systems are composed of multiple temperature-bound components with different *Q*_10_ values. In fact, robustness can be achieved only for specific component parameters and requires tight regulation. Moreover, not all activity attributes can be maintained simultaneously, thus, only the “most important” attributes can be temperature-robust^89^. Our results indicate that also in the larger and more complex networks of the central nervous system, such robustness can be maintained (Fig. 6). This is manifested by preserving state transitions and the abundance of each state while changing oscillation frequency. This may indicate which sleep property in *L. vulgaris* is more important: the presence of both states during each cycle rather than the cycle duration.

What are the limits of temperature robustness? We identified clear state transitions across a temperature range of 17–35 °C. Significantly, the temperatures we examined are those encountered during the summer in the natural habitat of *L. vulgaris*. The question remains - what happens to sleep states during the winter when night temperatures drop to 5 °C? This temperature would correspond to a *δ*/*β* period of ~21 min (Fig. 6f). One option is that brain states persist as long as lizards can find sleeping locations with adequate temperatures^90^, and that sleeping patterns stop completely during the cold part of the year. Correspondingly, we observed a slight increase in oscillation cycle variability at the edges of our temperature range, which may indicate state instability at temperatures higher than 35 °C or lower than 17 °C (Fig. 6p). If this is the case, will all cognitive sleep-dependent functions be impaired in extreme temperatures? As *L. vulgaris* (similarly to most lizards) hibernates during winter, most cognitive abilities may not be required. Interestingly, in mammals, sleeping patterns are mostly absent during hibernation at low temperatures (<20 °C) and thus hibernation is periodically interrupted for sleep^83,91^. Whether this is the case in reptiles awaits neurophysiological measurements from hibernating reptiles, but our results indicate that the lack of sleep during hibernation is not an outcome of low temperature as suggested for squirrels^91^. During spring and autumn in *L. vulgaris*’s natural habitat, temperatures are higher but can easily reach 10 °C and may thus still impair neurophysiological sleep patterns. Resolving this question awaits development of adequate behavioral paradigms and corresponding neurophysiological measurements that will allow us to link sleep patterns with behavioral impairment, or lack thereof.

Exploring the link between behavior and neurophysiology in reptiles may also help us understand the ancestral function of sleep states and guide us towards the fundamental functions of state transitions. Our findings suggest that repeated periodic transitions between active and silent states followed by a high continuous firing state are key features of sleep. Indeed, studies in humans highlight the importance of such periodic transitions in stabilizing memories^92,93^. In contrast to rodent model systems, sleep cycles in Agamid lizards are highly organized and can thus serve as a good model for studying the function of repetitive state transitions. Interestingly, the silent states during reptilian SWS are longer and the active states are shorter (~500 ms), much like the ShW in the hippocampus^10^. Thus, the ancestral sleep state may have manifested as short events that quickly propagate throughout the neural tissue^49,67,94,95^. Such propagation may therefore be a fundamental requirement of sleeping circuits that is fulfilled by up and down states in mammals^96,97^. To promote a comparative view of sleep and its functions, further work exploring the behavioral commonalities between lizard species exhibiting two-state sleep and mammals will be required.

## Methods

### Animals

Experiments were conducted on 9 adult (age unknown) rough-tail rock lizards (*Laudakia vulgaris*) from both sexes weighing 60–70 g. All animals were captured in the wild under the approval of the Israel Nature and Parks Authority (Approval number: 2021/42698). Once captured, lizards were moved to an animal house in Tel Aviv University’s zoological gardens and kept in a 12–12 h light (07:00–19:00) and dark cycle and an external temperature of 24 °C. The animal’s terrariums were equipped with a heat lamp (50 W), resulting in a local hot spot of 33 °C, and a UV light source that was active only during the day. Animals were provided with water ad libitum and fed with leafy green and a dose of protein in the form of mealworm and cockroaches covered in calcium and multi-vitamin powder. All experiments were approved by Tel Aviv University ethical committee (Approval number: 04-21-034).

### Surgery

Twenty-four hours before surgery, analgesics (Meloxicam: 0.2 mg/kg or Carprofen 2 mg/kg) and antibiotics (Baytril: 5 mg/kg) were administered. On the day of the surgery, the animal was initially anesthetized with inhalation of Isoflurane in an induction box and later intubated and connected to a ventilation system (AWS 100, Hallowell EMC) maintaining a constant flow of 4% Isoflurane. Once deep anesthesia was verified, the lizards were placed in a stereotactic apparatus (RWD 68409). Body temperature was maintained via a heating pad attached to the stereotactic table at 40 °C. Eyes were protected by covering with ointment (Doratears). The skin covering the skull was disinfected with Povidone Iodine 10% and coated with a Lidocaine ointment (2%) for local analgesia. The skin on the skull was removed with a scalpel and residual tissue was removed with a micro curette and dissolved with 30% hydrogen peroxide. A small craniotomy was drilled and the dura and arachnoid layers covering the forebrain were removed with fine forceps, and the pia was gently opened over the area of electrode insertion. A small incision in the brain was made with a 22 G needle lowered with a needle holder stereotaxic arm. A 32-channel flexible (4um thick) polyamide probe with TiN electrodes (custom fabricated by NMI, Germany) was inserted in the same location using a guide needle with manual adjustment. To target the DVR, probes were lowered to a depth of 1.5 mm below the cortical surface. The brain was then covered with Dora - gel (Cambridge neurotech). The remaining exposed skull was covered with UV glue (Transbond™ XT 3 M) and dental cement (Coral Fix) to secure the electrode and reference wire. Two holes were drilled contralaterally to the craniotomy and used for the placement of anchoring screws. The lizard was then removed from the stereotaxic table to a recovery box where it remained ventilated with Oxygen until it self-extubated. Following the surgery, the lizard received a daily dose (injected subcutaneously) of analgesics (Meloxicam: 0.2 mg/kg SC), antibiotics (Baytril: 5 mg/kg SC), and 1 ml of Saline for 5 days. Sleep recordings began after the lizard resumed normal behavior.

### Experimental paradigm

All experiments were performed in a specialized noise and temperature isolated room. The temperature in the room was controlled by an air conditioning system and monitored using a temperature sensor. At approx. 3 h before the beginning of the dark cycle (16:00), the animals were moved from their home terrarium to the behavioral room terrarium and were connected to the neurophysiological acquisition system. At approx. 3 h after the end of the dark period (10:00), animals were returned to their home cage. Light and dark cycles in the experimental arena were maintained as in the home cage (7:00–19:00).

### Electrophysiological recordings

Recordings were performed with an Open EPyhs acquisition system (acquisition board v2.2) and Intan amplifier head stages (RHD2132 - #C3324, or RHD2132 - #C3314 that included a 3-axis accelerometer) connected using an ultra-thin SPI cable (RHD2000-#C3216). The Open EPhys GUI^54^, was used for recording. Electrode recordings were referenced to a special on-probe reference electrode or an additionally implanted chlorinated silver wire (a-m systems, cat - 786000). During the recording, the weight of the Intan head stage was balanced using a pulley system.

### Video recording

In a subset of sleep nights, videos were recorded using a FLIR camera (Firefly DL) with an accompanying Moritex lens (ML-M1218UR). Images were collected using a custom script based on FLIR’s SDK “Spinnaker C++” at a rate of 50 fps or 10 fps. For synchronizing the recording and electrophysiology, frame time stamps were generated by Arduino Nano and sent to the camera and the Open Ephys data acquisition board. During the entire recording, the arena was illuminated with an IR projector. Video with missing frames (resulting in synchronization lags of more than 0.5 s) was rejected.

### Histology

After the experimental procedure was over, the lizard was deeply anesthetized with prolonged inhalation of Isoflurane in an induction chamber and decapitated with surgical sheers. The brain was then extracted with careful removal of bone and tissue with forceps and a micro-spatula and submerged in 4% PFA for 2 days. Afterward, the tissue was submerged in 15% sucrose in PBS solution for 24 h and then submerged for an additional 24 h in 30% sucrose in PBS solution. Following fixation, the tissue was embedded in optimal cutting temperature (OCT) embedding medium (Scigen - 4586) and was kept at −80 °C. The embedded brain tissue was sliced using a cryostat (Leica CM 1950) in 30 µm thick slices and Cresyl violet stained (Sigma - C5042). Stained brain slices were imaged using an Olympus epifluorescence microscope (model IX-81).

### Data analysis

Analysis was performed using custom scripts in Matlab (R2020b) or Python (3.9.0) unless specified otherwise.

### Spectral analysis of LFP

Spectral analysis was performed as in^48^. Briefly, voltage traces were low-pass filtered (100 Hz), downsampled (200 Hz), and binned (10 s). The average normalized power spectrum (spectrum in each bin divided by the average over the entire dataset) for each bin was calculated using the Welch method (1 s windows, 50% overlap), and the correlation matrix between all bin pairs (frequency range 0–30 Hz) was evaluated. The correlation matrix was separated into two groups using agglomerative clustering (ward linkage method over Euclidean distances). The intersection between the average (normalized) spectra of the two groups was defined as the transition frequency *F*_trans_. For calculating the *δ*/*β* ratio over the night, the extracted power spectra were calculated on 10 s bins (1 s steps). Based on the frequency transition values obtained before, we divided the mean non-normalized spectrum over frequencies lower than 4 Hz by the mean non-normalized spectrum over frequencies between 10 and 40 Hz in each bin.

### Extracting oscillation cycles, their P2V, and total sleep duration

Electrophysiological sleep duration was calculated using the autocorrelation of the *δ*/*β* ratio. First, the autocorrelation was calculated for a 4-h window during the center of the night. This gave an initial estimate of the periodicity in the recording. From this autocorrelation function, the global oscillation period and the global anti-phase lag were extracted by locating the first positive and negative non-zero peaks, respectively. Correspondingly, the global peak to valley (P2V) was defined as the difference between the autocorrelation’s first peak and first valley after subtracting two times the confidence value of the cross-correlation function (to indicate the value above this confidence bound). To evaluate the oscillation period dynamics, a floating autocorrelation was calculated on the *δ*/*β* time series (1000-s bins, 100-s steps) and smoothed using a moving average (10-s duration). For each bin, the local period and anti-phase lag were calculated by taking the maxima and minima during a 20-s interval surrounding the global period and global anti-phase lag (calculated before). This allowed identifying a consecutive period of sleep for each night. Sleep bins were defined as ones with P2V greater than 0.25 to avoid discontinuities due to brief decreases in autocorrelation amplitudes, the sleep time series was median filtered (1-h window) and binarized (threshold = 0.5) to indicate sleep or wakefulness. Since the global estimation of the oscillation period was calculated on a random section of the recording, we repeated this calculation on a 2-h window with the strongest oscillations evaluated by identifying the window with the highest P2V in the oscillation period dynamics function calculated above.

### Fitting temperature dependence

To extract the temperature scaling coefficient, we utilized the Q_10_ temperature coefficient formula to fit (least squares) *F*_0_ and *Q*_10_: $={F}_{0}{{Q}_{10}}^{\left(\frac{\left(T-{T}_{0}\right)}{10}\right)}$, where *F* denotes the sleep cycle oscillation frequency [1/min], ${F}_{0}$ the frequency at a temperature of ${T}_{0}$ = 18 °C and *T* the median temperature [°C] extracted from the ambient temperature logger.

### Timing of sleep cycles

The onset of the sleep oscillation cycles was calculated using the Hilbert transform over the sleep period (Fig. S2). First, the *δ*/*β* ratio was median filtered (20 s window) and Hilbert transformed. The instantaneous phase was extracted and its peaks were identified (findpeaks, Matlab) resulting in a series of timestamps of REM onsets. SWS onsets were defined as the time at which the Hilbert transforms between two consecutive REM onsets is minimum. Irregular cycles that were 1.5 times larger or smaller than the average cycle duration were removed from further sleep cycle analysis. We found this method to detect sleep cycles more robustly than the one previously used in *P. vitticeps*^48^.

### Respiration analysis

Respiration analysis (Fig. S3) was performed on videos that were synced with the electrophysiological recordings and included at least one long (>2 h) consecutive segment with minimal animal movements. The ribcage area was first manually marked by a rectangle and its position was tracked using the Kanade-Lucas-Tomasi (KLT) algorithm (pointTracker, Matlab). Points were detected using the minimum eigenvalue algorithm^56^. These points were re-detected every 100 s or on the following conditions: (1) the number of tracked points was <40, (2) there was a >10% decrease in the number of tracked points. The frames during which points were re-detected were not analyzed. The respiration time series (Fig. S3b), was defined as the first principal component (PCA, Matlab) of movement (over the x and y axes of the image) after applying a median filter (2 s window). To robustly extract the breathing rate (Fig. S3c), the autocorrelation function was used. First, the global respiration rate throughout the video was calculated by extracting the first peak (and valley) of the autocorrelation function (findpeaks, Matlab). Next, the respiration time series was binned (24 s windows with 50% overlap) and the first peak (in the segment [GP-2.5, GP + 5] where GP is the global peak), of the autocorrelation in each bin, was extracted to evaluate the local periodicity in respiration. Bins in which the valley lag exceeded the peak lag (which sometimes was the case in noisy segments) were rejected. The respiration rate was then resampled (as a function of cycle phase [0, 2π]), averaged over consecutive oscillation cycles and normalized between [0, 1] to evaluate the respiration dynamics as a function phase.

### Movement detection

Instances of body movement were extracted from the accelerometer attached to the Intan head stage (Fig. S4). Movements were detected along the X, Y, and Z-axis from the decimated (at 250 Hz after Chebyshev filter) accelerometer data. First, the difference between the upper and lower envelope was calculated using spline interpolation over local maxima (envelope, Matlab) and summed over all three axes. Micro-movements were detected as threshold crossings of the summed traces and their amplitude was recorded. The threshold was defined as 4 standard deviations above the noise level as calculated on noise segments. These segments were selected if their kurtosis (over 2 s bins) was ≤3. To quantify the degree of movements across sleep and wakefulness, the amplitude of supra-threshold movements was integrated over bins of 1 h. To identify the temporal distribution of micro-movements during SWS and REM, the number of micro-movements as a function of cycle phase [0, 2π] were counted for all oscillation cycles. For each night the average movement phase was calculated using CircStat ^55^.

### ShW detection and ripple analysis

The detection of ShW was based on a matched filter built from averaging 10,000 manually curated ShW from the recorded signals of different animals. Data was first low-pass filtered (scipy.signal, pass band 400 Hz) and resampled at 400 Hz (scipy.signal.resample) and then convoluted with the manually curated ShW template. The time of ShW events were extracted using peak detection (scipy.signal.find_peaks) over the convolved output with a height threshold of 0.25 (Fig. S1B). This number was chosen using an optimization scheme of F1-score over a labeled dataset with true and false ShW^57^. Nights with weak oscillations were rejected (P2V < 0.2) and events with detected artefacts (amplitudes > 1 mV) were removed. ShW autocorrelation and phase analysis was performed on binned (1.2 s) ShW rate vectors. For ripple spectral analysis, ShW were downsampled to 1000 Hz and the continuous wavelet transform (scipy.signal.cwt) with morlet wavelet was used to calculate the spectrogram. The amplitude in the ripple band (Fig. S1h) was extracted by calculating the Hilbert envelop over the bandpass-filtered (60-200 Hz butterworth) signals. This analysis was performed on recordings at 26–28 °C. Recordings were sorted according to decreasing signal quality (estimated by the averaged wide-band power over a 2 h epoch) and nights with average low power (<40 dB) were rejected. A total of 22 nights from 8 animals analyzed. Analysis of ShW across temperatures (Fig. 6) was performed on 71 nights in 9 animals. All ShW analysis was written in python 3.7.

### Eye movement analysis

Eye movements were calculated as in^48^ using the Lucas-Kanade method for optic flow estimation^58^ on tracked rectangular sub-frame segments surrounding the eye at a rate of 5 Hz. The position of the rectangle was manually annotated for the first frame in each video and tracked automatically throughout the rest of the night using the Kanade-Lucas-Tomasi (KLT) algorithm (pointTracker, Matlab) at an update rate of 0.6 Hz. The rectangle position used for optical flow calculations was updated if the tracked rectangle deviated from the current rectangle by more than 25% in either the vertical or the horizontal dimension. If the number of tracked points was smaller than 3 a new rectangle surrounding the eye was manually re-selected. The average optical flow was calculated by averaging the magnitude of all flow vectors in the rectangular frame (except for the frame edges, 5 pixels). Eye movements were considered significant only if their magnitude exceeded a floating threshold of 6 median absolute deviations (MAD) above the median calculated on a 3 min floating window. A shuffled control of these supra-threshold events was generated by replacing the event times within each cycle with ones drawn from a uniform distribution over the cycle duration.

### Statistics and reproducibility

Experiments were conducted until further experiments yielded no new knowledge. To reach this point, the number of experiments varied depending on the features tested and the difficulty of the experiment. Sample sizes are specified for each figure and defined accordingly to animal, recorded night or sleep cycles. Experiments focus on measurements from animals implanted with electrodes. Animals that died during the implantation procedure or ones for which the implantation procedure failed, were not included in the analysis. Other exclusions were made on the basis of stringent criteria, which are specified in the relevant method section. In cases where videos were analyzed, only videos with reliable syncing with electrophysiological data were included.

### Reporting summary

Further information on research design is available in the Nature Portfolio Reporting Summary linked to this article.

## Supplementary information

Supplementary information

Description of Additional Supplementary Data

Supplementary video 1

Supplementary video 2

Reporting Summary-New

## Data Availability

The datasets generated during and/or analyzed during the current study are available from the corresponding author on reasonable request. Source data for the figures can be found at https://github.com/EvolutionaryNeuralCodingLab/Temperature-robust-REM-and-SWS-in-Laudakia-vulgaris.

## Code availability

The code used to analyze the data can be found at https://github.com/EvolutionaryNeuralCodingLab/Temperature-robust-REM-and-SWS-in-Laudakia-vulgaris.
