# Supplementary material for: Temperature-robust rapid eye movement and slow wave sleep in the lizard Laudakia vulgaris
Source: Commun Biol. 2022 Nov 29;5:1310. doi: 10.1038/s42003-022-04261-4 (PMC9709036; doi:10.1038/s42003-022-04261-4)
Supplement: Supplementary file 1 — Supplementary information [file 42003_2022_4261_MOESM1_ESM.pdf]

Temperature-robust rapid eye movement and slow wave sleep in the lizard *Laudakia vulgaris*

N. Albeck[1,2,†], D. I. Udi[1,†], R. Eyal[1,2] and A. Shvartsman[1] and M. Shein-Idelson[1,2]
[1]School of Neurobiology, Biochemistry, and Biophysics, Tel-Aviv University, Israel
[2]Sagol School of Neuroscience, Tel-Aviv University, Israel, †Equal contribution

Supplementary information

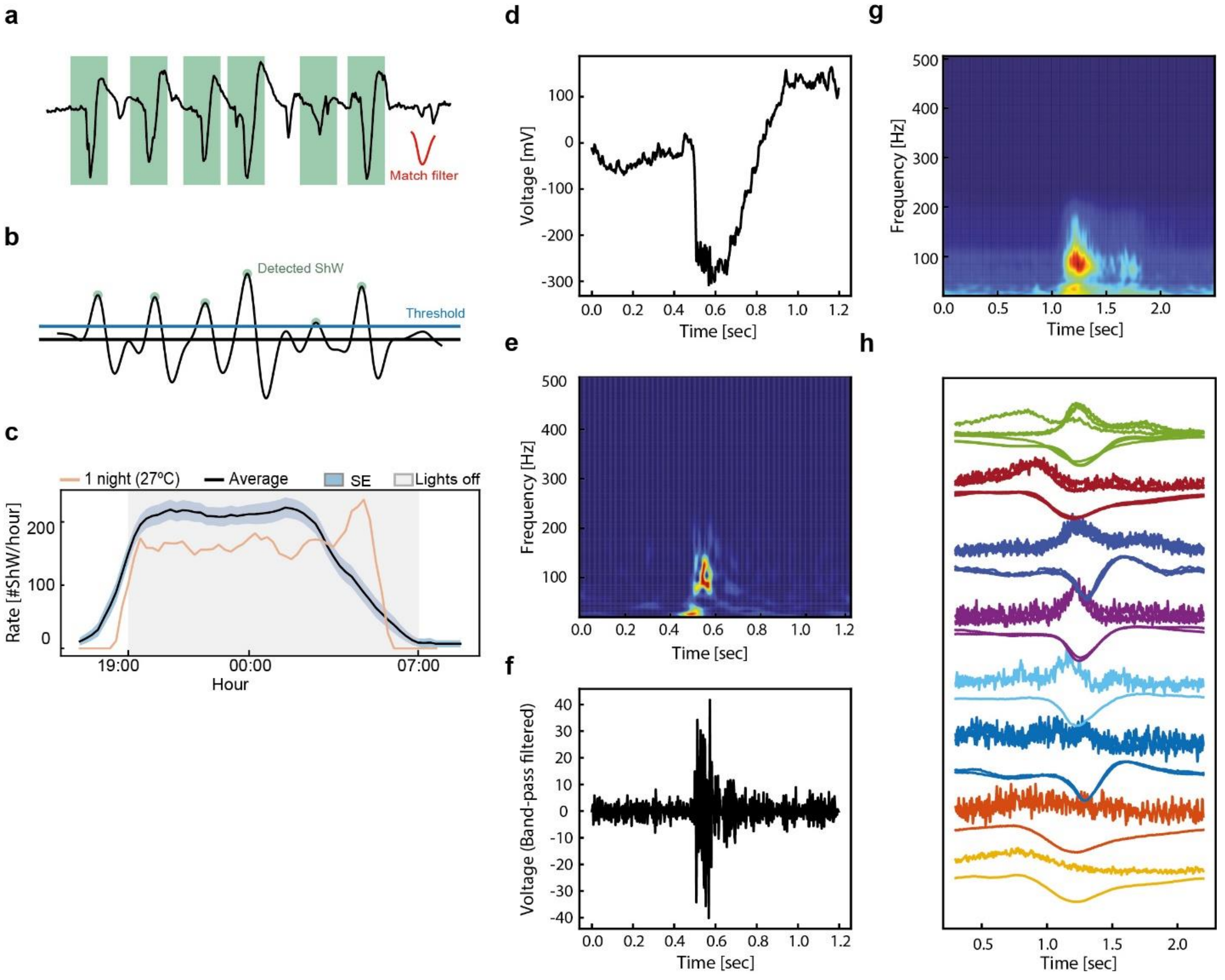

Figure S1. ShW detection and characteristics during sleep. (a) Detection of ShW. An example of a down-sampled (50Hz) signal containing ShWs is shown in black. Green rectangles (Height=1mV, width=800ms) mark detected ShW. (b) ShW detection method. Black: The electrode signal is convolved with a matched filter (red curve). ShW, marked in green circles, detected as peaks larger than threshold (blue line, see methods). (c) The rate of ShWs during an entire recording. A single night at 27ºC (orange), and an average over all recordings (black). Blue shades mark the standard error. Grey shade marks the lights off period. (d) Example raw data trace of a sharp wave detected by the algorithm. (e) Spectrogram for the ShW in (d) extracted using the continuous wavelet transform (morlet wavelet). This spectrogram shows an increase in power for the 80-200Hz band at the trough of the ShW. (f) A bandpass filtered (60-200Hz butterworth) trace of the data in (d) showing the increase in power during the ShW. (g) Average spectrogram (calculated as in (e)) of all ShWs from nights recorded at 26-28ºC, 22 nights from 8 animals, showing a transient increase in 80-200Hz power. (h) The normalized (0-1) average Hilbert envelope of the bandpass-filtered (80-200Hz) ShWs traces (as in f) together with the average ShW trace (lower traces for each recording). Results are grouped by animal and ordered along the vertical axis by decreasing recording quality from top to bottom (defined as the average total power calculated on a 2h segment of each sleep recording). 22 night from 8 animals, color code as in figure 3j.

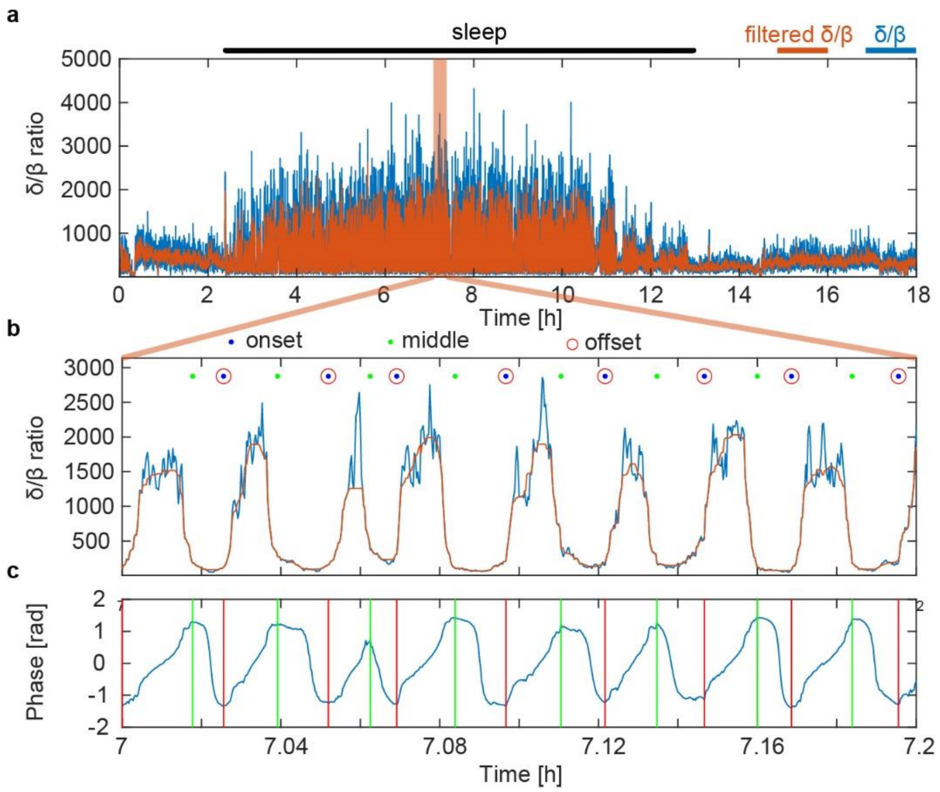

Figure S2. Detection of oscillation cycles based on the Hilbert transform. (a) δ/β dynamics throughout the night (blue - δ/β, red - median filtered δ/β). (b) Zoom into a small segment in (a). Blue dot, green dots, and red circles correspond to onsets of SWS, onsets of REM, and offsets of REM, respectively. (c) The instantaneous phase calculated from the Hilbert transform on the trace in (b). Notice that phase peaks and valleys correspond to the beginnings and endings of REM segments (low δ/β values). Red lines correspond to the onsets of SWS and offsets of REM states (see also blue dots and red circles in (b)). Green lines correspond to the onsets of REM.

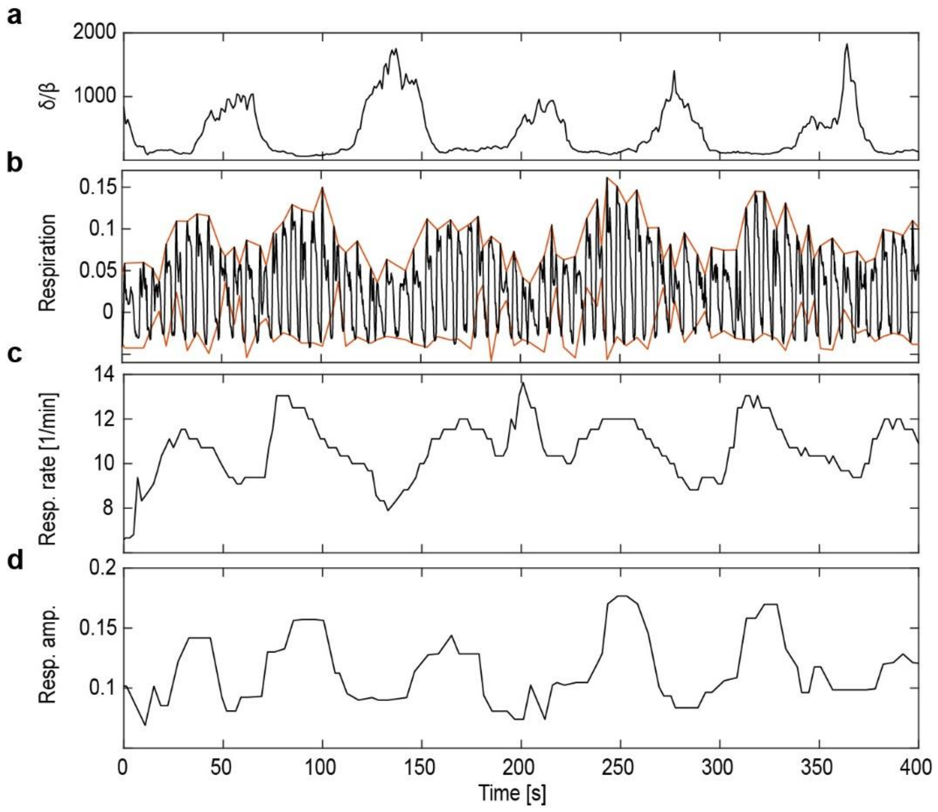

Figure S3. Video respiration analysis. (a) δ/β fluctuations recorded during sleep. (b) Respiration signal (black) extracted from the ribcage movement using video analysis during the time segment in (a) (see Methods and Video S2) and its envelop (orange). (c) Respiration rate calculated for the signal in (b) using the floating auto correlation function peak. (d) Respiration amplitude extracted from the signal envelope (calculated on the signal in (b)). Notice respiration rate and amplitude increases during REM.

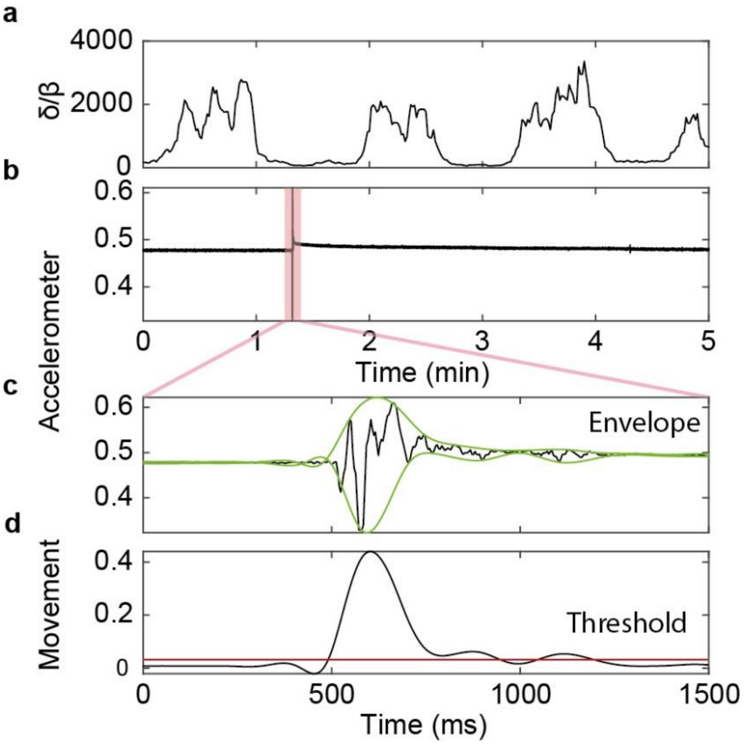

Figure S4. Micro-movement calculation. (a) δ/β dynamics (see Figure S2) over a small segment of the recording. (b) The simultaneously measured accelerometer trace from one axis during the same time period as in (a). (c) Zoom into the accelerometer trace (during the red segment marked in (b)). Green traces mark the envelope. (d) The movement signal is defined as the envelope height (black trace) calculated on the data in (c). The threshold (4 std above noise level) is marked in red and use for event detection. Only above threshold movement points are considered in the analysis.

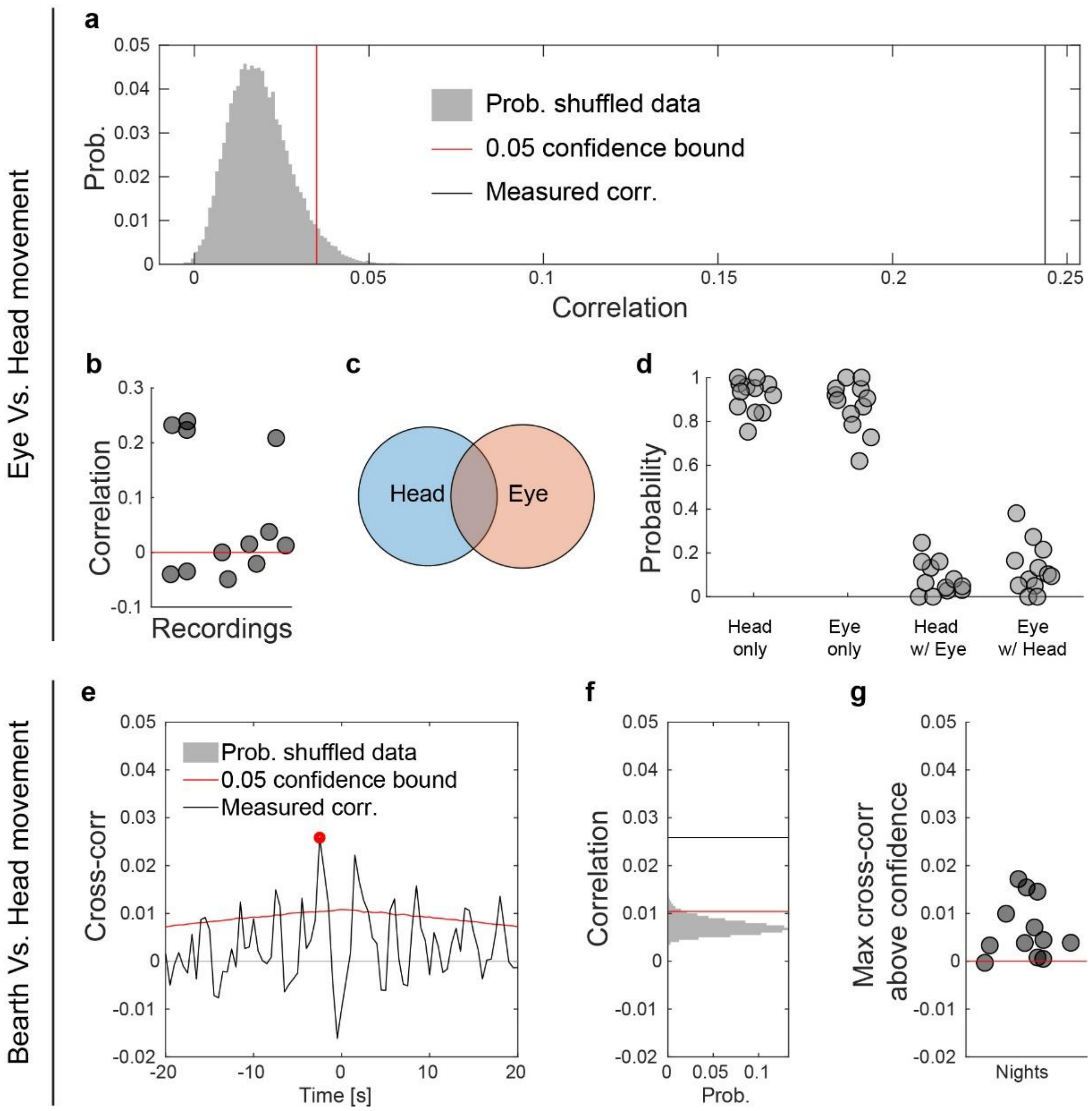

Figure S5. Head micro-movements are mildly but significantly correlated with eye movements and breathing. (a) Pearson correlation between eye and head movement during REM segments for one night (black vertical line), compared to the distribution of correlation values for shuffled data (n=50,000, gray). The 0.05 confidence correlation value is marked by a red vertical line. (b) The correlation above the 0.05 confidence bound (red line) for all analyzed experiments (12 nights from 4 animals). In 4 recordings, this correlation was much higher than the confidence bound. (c) Vann diagram depicting the fraction of co-occurring head and eye movements, for the night presented in (a). While some eye-head movements co-occur, most do not. (d) Probabilities for co-occurring and non-co-occurring eye and head movements for all recorded nights. (e) cross-correlation between breathing and head movements. The maximum cross-correlation is marked by the red dot and the 0.05 confidence bound for each time lag by a red line. (f) The maximal value (black line) of cross-correlation compared to the maximum of the shuffled data (gray). The 0.05 confidence bound determined by shuffling is marked by the red line. (g) Correlation above the confidence bound (red line) for the maximal cross-correlation for all nights (gray dots). While these values are significantly higher than for randomly shuffled data, they are very low suggesting limited interaction between breathing and head movement. See supplementary methods for more details.

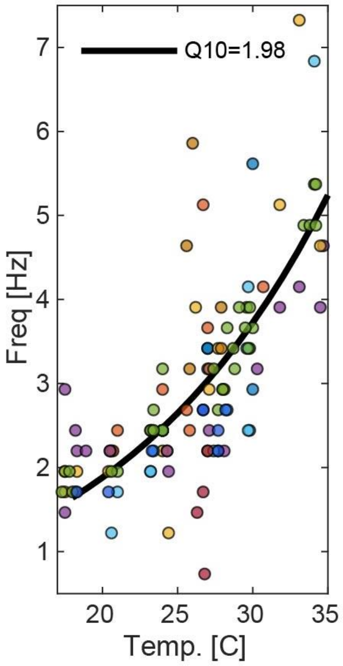

Figure S6. Transition frequency between SWS and REM increases with temperatures. The transition frequency between spectral profiles (as calculated in Fig 3g) for all nights and different temperatures (as in 6f). Fitting the data to the function $F_{Trans}=F_0Q_{10}(T-T_0)/10$ (black line, as in Fig 6f) resulted in a Q_{10} coefficient of 2.0.

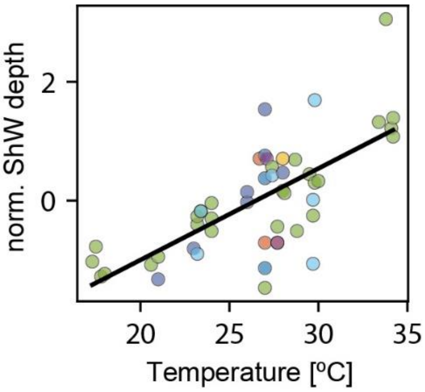

Figure S7. ShW amplitude increases with temperature. ShW amplitude values (difference between minimum and maximum) were detected from the average ShW shapes for each recording (as in Figure 6o). The amplitude is plotted for 50 nights and different colors correspond to different animals. Color code as in Figure 3j. Correlation Coefficient=0.7, t-test, P-Value=$2e^{-8}$.

Supplementary Methods

To assess the interaction between head micro-movements, eye movements and breathing movements, we performed correlation analysis on the experiments in which eye video, breathing video and accelerometer movements were recorded synchronously. First, eye and head movements were extracted as previously described (Figure 5a-d). Next, their time series were concatenated, binned (bin size = 1s) and the Pearson correlation between segments of both time series was calculated. The correlation value was compared with the correlation after shuffling the intervals between eye movements (n=50,000 randomizations) to evaluate the P-Value. Figure S5a shows the probability distribution of the correlation function (gray), the 0.05 confidence interval (red line) and the measured correlation value (black line) for one experiment. Significant (P-Value<2e-5) co-occurrence of head and eye movements is evident for this recording. Figure S5b shows the correlation value above the 0.05 confidence interval for all analyzed experiments (12 nights from 4 animals). In about half of the recorded nights a significant correlation was observed (Figure S5b) and in 4 nights it had high correlation values. To estimate how frequent were these co-occurrence events, co-occurrence was estimated by calculating the fraction of bins in which eye and head movements are found in the same bin. This is illustrated in the Vann diagram for one night (Figure S5c - same night as in Figure S5a). Across all recorded nights, the large majority of head and eye movements occurred independently. We emphasize that this correlational analysis does not determine the causality between events. Thus, eye and head movements could be driven by the same neurophysiological process or may be the result of measurement coupling. For example, movement of the eye can register on the accelerometer if it was strong enough to elicit a head movement. In this case, we would expect eye movements and head movements to be significantly correlated in intensity. This was not the case for 83% of analyzed recordings. In addition, since eye movements largely occur without head movements and vice-versa, these movements are likely driven by independent but potentially coupled sources. The above analysis was conducted only on the REM state since most of the movements occurred in this state. Including all sleep states in the analysis did not qualitatively change the results.

Quantifying the interaction between breathing and head movements was more challenging since breathing is a continuous process, thus by nature, breathing movements always occur during head movements. We therefore used cross-correlation analysis in order to examine if the local breathing periodicity was reflected in head movements. We first binned head movements (bin size = 0.5s) and cross-correlated (Figure S5e) them with the breathing times defined as the middle between every two consecutive breathing peaks (see Figure S3). From this cross correlation, we identified the maximum (Figure S5e - red dot) and calculated its significance against 50,000 randomly shuffled time series for which the maximum value was similarly calculated (Figure S5f). Doing so for a single night, shows that breathing movements were

significantly correlated with head movements. Such a significant correlation (above a confidence band of 0.05) was evident in most recordings (Figure S5g; 12 nights from 4 animals). However, while significant, these maximal cross-correlation values in all recordings were very low (<0.02). This suggests that the contribution of breathing related movements to head movements is minor.

Supplementary video 1. Eye movement analysis during sleep. A video acquired during sleep (sped up X10). Top left panel: δ/β ratio as a function of time. Top right: changes in Optic flow as a function of time, using the Lucas-Kanade method for optic flow estimation (see Methods).

Supplementary video 2. Breathing analysis during sleep. A video acquired during sleep (sped up X4). Markers on the lizard's ribcage (pluses) are tracked using the Kanade-Lucas-Tomasi algorithm (see Methods).
