# Supplementary material for: Temperature-robust rapid eye movement and slow wave sleep in the lizard Laudakia vulgaris
Source: Commun Biol. 2022 Nov 29;5:1310. doi: 10.1038/s42003-022-04261-4 (PMC9709036; doi:10.1038/s42003-022-04261-4)
Supplement: Supplementary file 2 — Description of Additional Supplementary Data [file 42003_2022_4261_MOESM2_ESM.docx]

**Description of Additional Supplementary Files**

**File name:** Supplementary Video 1

**Description:** Eye movement analysis during sleep. A video acquired during sleep (sped up X10). Top left panel: δ/β ratio as a function of time. Top right: changes in Optic flow as a function of time, using the Lucas-Kanade method for optic flow estimation (see Methods).

**File name:** Supplementary Video 2

**Description:** Breathing analysis during sleep. A video acquired during sleep (sped up X4). Markers on the lizard’s ribcage (pluses) are tracked using the Kanade-Lucas-Tomasi algorithm (see Methods).
